# WFDC2 suppresses prostate cancer metastasis by modulating EGFR signaling inactivation

**DOI:** 10.1038/s41419-020-02752-y

**Published:** 2020-07-16

**Authors:** Yaoyi Xiong, Lushun Yuan, Song Chen, Huimin Xu, Tianchen Peng, Lingao Ju, Gang Wang, Yu Xiao, Xinghuan Wang

**Affiliations:** 1 Department of Urology, Zhongnan Hospital of Wuhan University, Wuhan, China; 2 Division of Nephrology, Department of Internal Medicine, Leiden University Medical Center, Leiden, The Netherlands; 3 Department of Biological Repositories, Zhongnan Hospital of Wuhan University, Wuhan, China; 4 Human Genetics Resource Preservation Center of Hubei Province, Wuhan, China; 5 Laboratory of Precision Medicine, Zhongnan Hospital of Wuhan University, Wuhan, China; 6 Medical Research Institute, Wuhan University, Wuhan, China

**Keywords:** Prostate cancer, Prostate cancer

## Abstract

WAP four-disulfide core domain 2 (WFDC2) is a small secretory protein that has been widely studied in ovarian cancer. It has been proven that WFDC2 promotes proliferation and metastasis in ovarian cancer, and serves as a diagnostic biomarker. However, the specific function of WFDC2 in prostate cancer has not been reported. Here, we first screened the diagnostic marker and favorable prognostic factor WFDC2 in prostate cancer by bioinformatics. WFDC2 expression was negatively correlated with Gleason score and metastasis in prostate cancer. Then, we revealed that overexpression of WFDC2, and addition of recombinant protein HE4 can significantly inhibit prostate cancer metastasis in vivo and in vitro. By co-immunoprecipitation and co-localization assays, we proved that WFDC2 binds to the extracellular domain of epidermal growth factor receptor (EGFR). Immunoblot showed that WFDC2 overexpression and recombinant protein HE4 addition inactivated the EGFR/AKT/GSK3B/Snail signaling pathway, and then restrained the progression of epithelial–mesenchymal transition. In conclusion, our study identified that the tumor suppressor WFDC2 can suppress prostate cancer metastasis by inactivating EGFR signaling.

## Introduction

Prostate cancer has the highest incidence of any malignancy in men in many regions worldwide. Approximately 1.3 million new cases were diagnosed in 2018, accounting for 13.5% of all cancer diagnoses^1^. Most early-stage prostate cancer has a satisfactory survival time after surgery and/or androgen deprivation therapy (ADT)^2^. Once primary prostate cancer progresses to metastatic prostate cancer, patients have a poor outcome.

Metastasis is the leading cause of death in the majority of patients with cancer, including prostate cancer^3^. Many studies have attempted to decipher the mechanism of tumor metastasis^4–6^. The relationship between epithelial-to-mesenchymal transition (EMT) and tumor metastasis is the most studied topic in this field. The EMT process promotes the polarization of epithelial cells and imparts mesenchymal cell properties, which enhance the ability of cell migration and invasion. Therefore, the activation of EMT is considered the key process in the development of tumor metastasis^7^.

WAP four-disulfide core domain 2 (WFDC2), which encodes the small secretory protein human epididymis protein 4 (HE4), is widely upregulated in ovarian cancer^8,9^. At present, HE4 serves as a serum biomarker of ovarian cancer, and has better sensitivity and specificity than CA125^10^. However, the application of HE4 in clinical diagnosis has attracted more attention than its specific function. Several papers have revealed that HE4 is correlated with the metastasis of ovarian cancer^11,12^. Interestingly, we found that WFDC2 is downregulated in prostate cancer compared to ovarian cancer. This raised our interest regarding the function of WFDC2 in prostate cancer.

In our study, we identified that the tumor suppressor WFDC2 can obviously inhibit the metastasis of prostate cancer in vitro and in vivo. We further proved that WFDC2 binds to the extracellular domain of epidermal growth factor receptor (EGFR). Therefore, we speculate that WFDC2 inhibits prostate cancer metastasis by inactivating EGFR signaling.

## Materials and methods

### Cell culture and reagents

Human prostate cancer PC-3 and DU-145 cell lines were cultured in RPMI-1640 with 10% fetal bovine serum (FBS). The 293 T cell line was cultured in DMEM with 10% FBS. PC-3, DU-145, and 293 T cell lines were obtained from the American Type Culture Collection and were recently authenticated.

Antibodies against FLAG (F1804, Sigma), HA (TA180128, OriGene), GAPDH (sc-365062, Santa Cruz), WFDC2 (ab200828, Abcam), EGFR (ab52894, Abcam), p-EGFR (4407 S, CST), AKT (4691 L, CST), p-AKT (4060 L, CST), c-Myc (ab32072, Abcam), E2F-1 (ab179445, Abcam), GSK3B (12456 S, CST), p-GSK3B (5558 S, CST), Snail (3879 S, CST), E-cad (3195 S, CST), N-cad (13116 S, CST), Vimentin (5741 S, CST), and secreted protein HE4 (CSB-DP018B, CUSABIO), EGF (PHG0311L, Gibco) were purchased from indicated commercial sources.

### Plasmid construction and transfection

The primer used to amplify human WFDC2, EGFR-FL, EGFR-NT, and EGFR-CT was listed in Supplementary Table 1. WFDC2 cDNA was subcloned into a pcDNA5-HA vector. EGFR-FL, EGFR-NT, and EGFR-CT cDNA were subcloned into a pcDNA3.1-FLAG vector. The siEGFR: 5′-GCGUUAGACUGACUUGUUUTT-3′ was purchased from Shanghai GenePharma Co., Ltd. Before transfect into cells with Lipofectamine 3000 (Invitrogen), the recombinant plasmid was sequenced to confirm the accuracy without mutation.

### RNA isolation, reverse transcription, and qRT-PCR

Total RNA was isolated using RNeasy Mini Kit (cat. #74101, Qiagen) according to the manufacturer’s instruction. The cDNA was synthesized using 1 μg of total RNA and quantitative real-time PCR (qRT-PCR) was performed using 500 ng cDNA per 20 μl reaction. Each reaction was conducted with iQTM SYBR® Green Supermix (Bio-Rad) using 500 ng of cDNA in a final volume of 20 μl. Primer sequences and annealing temperatures are summarized in Supplementary Table 2. Values were normalized for amplified GAPDH alleles.

### Xenograft mouse model

PC-3-GFP vector and PC-3-GFP WFDC2 overexpressing virus were purchased from Shanghai GenePharma Co., Ltd. Then, the PC-3 cells were infected with virus and the positive cells were selected with 1 μg/ml puromycin (Sigma). Male NOD/SCID mice were obtained from Beijing HFK Bioscience Co., Ltd. Mice were randomly divided into two group (*n* = 2). A total of 1 × 10^6^ PC-3 LV-WFDC2 or LV-vector cells diluted in 0.1 ml of PBS were injected into the tail vein of 8-week-old NOD/SCID mice. Metastasis was monitored by fluorescence detection of GFP-expressing cells and formation of bone lesions by X-ray. After growth for another 6 weeks, Living Image software (Caliper Life Sciences) was used to quantify the fluorescence intensity. We are blinded to the group allocation when assessing the fluorescence intensity, all experimental protocols were approved by the Wuhan University Institutional Animal Care and Use Committee.

### Transwell assay

The polycarbonate transwell filters (Corning) was placed in 24-well plates with 0.2 ml culture media without FBS. A total of 5 × 10^4^ cells were seeded in upper chamber. After incubation for 24 h, cells were fixed with 4% paraformaldehyde and stained with crystal violet.

### Wound healing assay

A total of 1000 cells were seeded in six-well plates. When the cell fusion reaches 100%, we scratched the cells with a 200 μl pipette tip. After washed with PBS, the cells were incubated in culture medium without FBS. Then the cells were photographed with microscope after incubation for 24 h.

### MTT assay

The MTT (methyl thiazolyl tetrazolium, Sigma) assay was used for cell viability measurement in PC-3 and DU-145 cells. After transfection for 48 h, PCa cells were seeded in 96-well plates (3000 cells per well) in RPMI-1640 medium containing 10% FBS for 5 days. Then, 20 μl of MTT reagent was added to each well for 4 h at 37 °C. After discarding the medium, the precipitates were dissolved by 200 μl of DMSO. The absorbance was measured at 490 nm using a Spectramax M5 spectrophotometer (Molecular Devices).

### Flow cytometry analysis

For cell cycle analysis, PC-3 and DU-145 cells were harvested and washed with cold PBS three times after transfection for 48 h. Then, the cells were resuspended in 1× DNA Staining Solution containing propidium iodide and permeabilization solution (Multisciences) in the dark. After incubation at 37 °C for 30 min, the samples were analyzed by flow cytometry (cat. #FC500, Beckman).

### Immunoblot assay

The cells were lysed on ice for 30 min using a mixture of phosphatase inhibitor, protease inhibitor, and RIPA buffer. The supernatant was collected after centrifugation at 14,000 × *g* for 10 min at 4 °C. Then, the protein concentration was measured by bicinchoninic acid (BCA) assay. Protein extracts were isolated by SDS–PAGE gel and then transferred to a PVDF membrane. The membrane was then blocked in TBS-Tween buffer containing 5% skim milk, and incubated sequentially with primary and secondary antibodies. An enhanced chemiluminescence kit was used to expose the bands.

### Immunoprecipitation assay

Twenty microliters of Protein A magnetic beads were incubated with 1 μg of the target antibody for 4 h at 4 °C. After washing twice with Triton X-100 buffer (150 mM Tris, 150 mM NaCl, 0.4% NP-40, pH 7.4), whole cell lysates were added to the antibody–bead complex and incubated overnight at 4 °C. Subsequently, the cells were washed four times with Triton X-100 buffer. The protein–antibody–bead complex was then eluted with 1× SDS buffer for further immunoblot analysis.

### Immunofluorescence staining

A total of 1 × 10^5^ cells were plated overnight in a six-well plate containing cell slides. The next day, the cells were fixed with 4% formaldehyde for 20 min at room temperature (RT). Subsequently, the cells were washed three times with PBS and incubated with buffer (2% BSA plus 0.3% Triton X-100) for 1 h at RT. In addition, cells were incubated for 2 h at 4 °C in the corresponding primary antibody. The cells were washed three times with PBS and then incubated with the secondary antibody for 2 h at RT. After incubation, cells were washed with PBS and incubated with DAPI (1:1000) for 5 min at RT. After staining the nuclei, the cells were sealed and air-dried overnight and then photographed on a 60× oil mirror on a confocal microscope.

### Dataset collection

The GSE70770, GSE116918, GSE3325, and GSE8511 datasets were downloaded from the GEO database (https://www.ncbi.nlm.nih.gov/geo/), the MSKCC PRAD dataset was downloaded from the cBioPortal database (https://www.cbioportal.org/), and TCGA-PRAD pan-cancer normalized data were downloaded from the UCSC Xena database (https://xena.ucsc.edu/). The GSE70770 dataset was used as the training set for co-expression network construction, prognostic value validation, and functional prediction analysis. GSE116918, MSKCC PRAD, and TCGA-PRAD were used as the validation sets for prognosis and functional analysis. Furthermore, GSE3325 and GSE8511 were used to validate the metastasis-related phenotype. Data were analyzed with the R (version 3.5.2) and R Bioconductor packages.

### WGCNA construction and identification of prostate cancer diagnosis-related modules

The weighted gene co-expression network, “WGCNA”, R package was used to construct co-expression network, we first calculated the standard deviation values for gene expression in GSE70770, ranked by it and chose the top 25% for further analysis^13,14^. Outlier samples were checked and removed. Then, the proper soft-thresholding parameter *β* was chosen, and genes with similar expression patterns were clustered into the same module to construct the scale-free network. By combining with the clinical information, including PSA value, total Gleason score, primary Gleason score, secondary Gleason score, and tumor percentage (%), we identified modules and genes associated with clinical information characteristics.

### Diagnostic value validation via public database and tissue microarray

To further validate our concerned gene, the Oncomine database (https://www.oncomine.org/) and Human Protein Atlas database (https://www.proteinatlas.org/) were used for transcriptional validation and translational validation^15,16^. The tissue microarray, purchased from Shanghai Outdo Biotech, contained 95 prostate cancer tissues (including 11 with Gleason 3 + 3, 29 with Gleason 3 + 4, 14 with Gleason 4 + 3, 13 with Gleason 8, 21 Gleason 9, and 7 with Gleason 10) and five non-tumor tissues (including three normal and two paracancerous prostate tissues). Briefly, paraffin sections were deparaffinized first, then antigen retrieval was performed in citrate buffer (pH 6.0), and endogenous peroxidase activity was blocked in 0.3% H_2_O_2_. Subsequently, all slides were incubated with primary and secondary antibodies until visualization by peroxidase and 3, 3′-diaminobenzidine tetrahydrochloride. The expression of WFDC2 in the prostate tissues from the tissue microarray was blindly quantified by two pathologists. Immunohistochemical sections were analyzed using a phase-contrast microscope and the staining intensity was defined as negative, 1, 1–2, 2, 2–3, or 3. Furthermore, receiver operating characteristic (ROC) curves were generated to demonstrate the role of WFDC2 in distinguishing different Gleason score for PCa and non-tumor tissues^17^.

### GSEA and GSVA

Based on TCGA-PRAD pan-cancer normalized data, we chose the median expression of WFDC2 as the cutoff and divided the samples into high/low expression groups. Then, we chose differently expressed metastasis/EMT-related gene sets for the metastasis/EMT phenotype and the differently expressed gene sets between PCa vs non-tumor for the PCa diagnostic evaluation. The analysis was performed and visualized by javaGSEA (gene set enrichment analysis) or clusterProfiler. *p* Value and FDR < 0.05 were chosen as the cutoff^18–20^. For the gene set variation analysis (GSVA) analysis, we chose HALLMARK gene sets as the reference, *t* value > 2 and *p* value < 0.05 as the cutoff to screen significantly altered pathways^21^.

### Survival analysis

All prognostic information from GSE70770, GSE116918, MSKCC PRAD, and TCGA-PRAD were collected, and samples without outcome information were removed. Based on the optimal separation of samples, Kaplan–Meier (KM) survival curves were generated to calculate survival rates (recurrence-free survival (RFS), metastasis-free survival (MFS), and disease-free survival (DFS)), the significance of differences between survival curves was determined using the log-rank test and visualized through the “forestplot” package in R.

### Statistical analyses

All analyses were performed at least three times and represented data from three individual experiments. Two-tailed Student’s *t*-tests were used to assess the statistical significance of differences between the groups. Statistical analyses were performed using SPSS 16.0. Statistical significance was considered as a *p* value < 0.05.

## Results

### Identification WFDC2 as a PCa diagnostic marker via WGCNA analysis

After filtering by the standard deviation value cutoff, we finally identified 5234/20,933 genes for further co-expression network construction. No samples were removed (Supplementary Fig. 1) and sample clusters with clinical information were shown in Supplementary Fig. 2a. To ensure a scale-free network, *β* = 12 was chosen as the proper soft-threshold and then a dynamic tree cut was built (Supplementary Fig. 2b, c). Combined with the module trait relationship and module significance, we eventually identified the red module as the module most correlated with tumor diagnosis (Supplementary Fig. 2d, e). Interestingly, WFDC2 was a member of the red module, which was highly negatively correlated with tumor percentage (%).

### WFDC2 is dramatically downregulated in human prostate cancer and negatively correlated with Gleason score

**Fig. 1:**
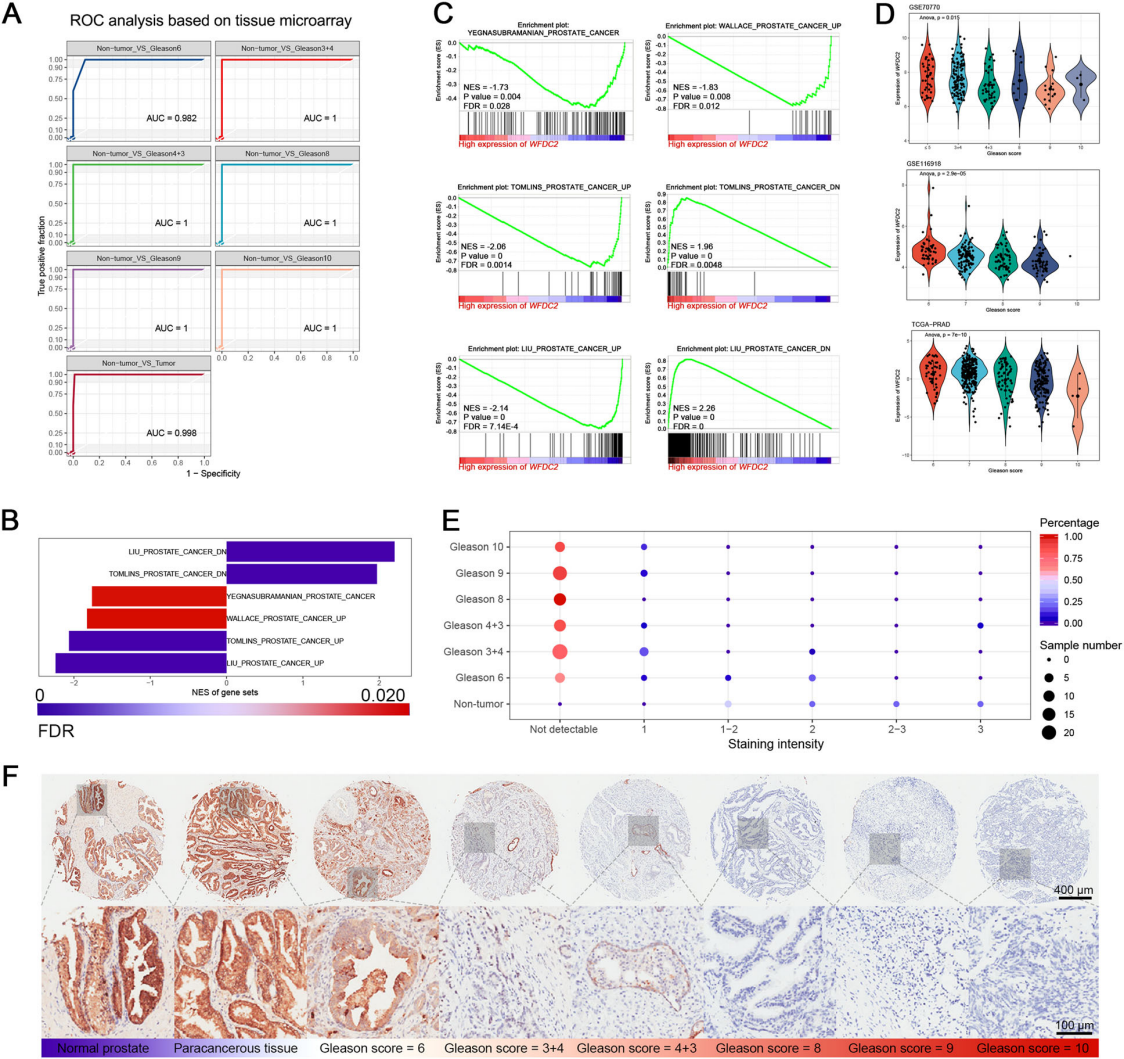
High expression of WFDC2 has tropism toward a non-prostate cancer phenotype. **a** ROC analysis for distinguishing non-tumor tissues from prostate cancer tissues based on tissue microarray. **b**, **c** Barplot of GSEA analysis and separated gene sets for the diagnostic role of WFDC2. **d** The expression of WFDC2 by different Gleason score based on GSE70770, GSE116918, and TCGA-PRAD. **e** Dotplot showing the overview of the tissue microarray. The color of the bar represents the percentage of tissue samples in each sample type and the size of the dot represents the sample number. **f** HE staining (top panel) and IHC staining (middle and bottom panel) of the tissue microarray. The scale bar of HE staining and IHC staining (middle) is 400 μm and the scale bar of IHC staining (bottom) is 100 μm.

WFDC2 was first identified as a diagnostic marker by co-expression network analysis. To further validate its diagnostic value, we performed validation at the transcriptional and translational levels. We found that WFDC2 was an oncogene in several cancers using the Oncomine database, but as a tumor suppressor in PCa, which was proven by ten independent datasets (Supplementary Fig. 2f). We observed that WFDC2 expression was significantly downregulated in tumor tissues compared with non-tumor tissues (Supplementary Fig. 2g). Moreover, we also obtained the same trend from the Human Protein Atlas database. WFDC2 was strongly upregulated in normal prostate tissues and could not be detected in prostate cancers (Supplementary Fig. 3). Meanwhile, based on the GSEA analysis, high expression of WFDC2 samples had a tropism toward non-prostate cancer (Fig. 1b, c). Then, we performed ANOVA in PCa samples with different Gleason scores, it was surprising that the expression of WFDC2 significantly decreased with increasing Gleason scores (Fig. 1d). To further validate our hypothesis, a tissue microarray was performed, and we found that WFDC2 was dramatically downregulated in PCa that could not be detected (Fig. 1e, f). ROC analysis showed that the area under the curve for the Gleason score of non-tumor vs PCa samples was close to 1, representing its strong potential for distinguishing non-tumor samples from PCa samples (Fig. 1a). The clinical information of the tissue microarray was listed in Supplementary Fig. 4.

### WFDC2 has no effect on the proliferation and apoptosis of prostate cancer

We performed MTT assay and flow cytometry analysis to explore the specific biological role of WFDC2 in PCa. Compared to the vector group, the WFDC2 upregulated group had no obvious effect on the proliferation of PCa (Supplementary Fig. 5a, b). For cell cycle and apoptosis, overexpression of WFDC2 also had limited effects (Supplementary Fig. 5c–f).

### Overexpression of WFDC2 suppresses prostate cancer metastasis in vivo and in vitro

**Fig. 2:**
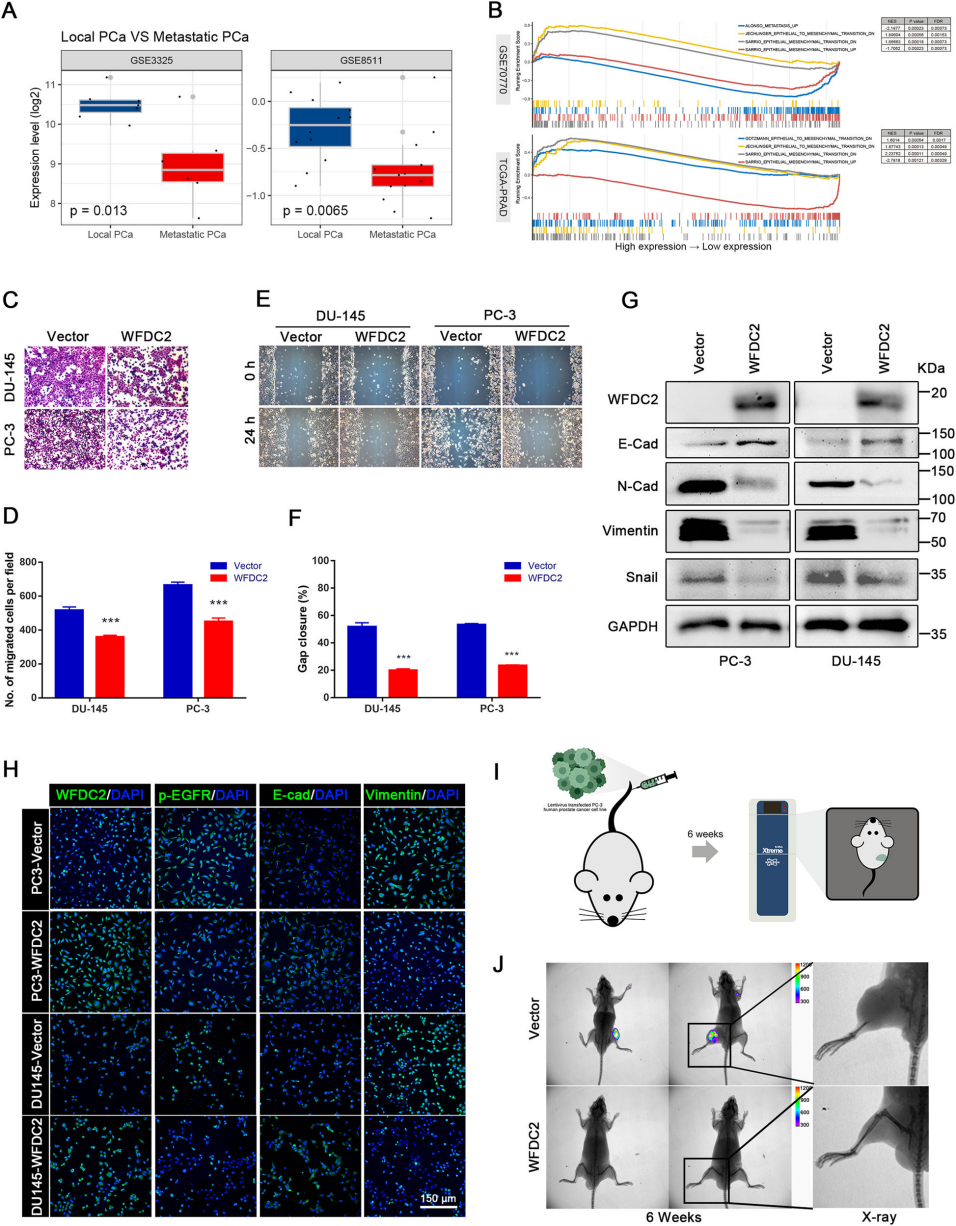
Overexpression of WFDC2 suppressed prostate cancer metastasis in vivo and in vitro. **a** The expression of WFDC2 in GSE3325 and GSE8511. **b** The relationship between WFDC2 and EMT analyzed by GSEA. **c**, **d** Transwell assay in DU-145 and PC-3 after WFDC2 upregulation. The scale bar is 150 μm. **e**, **f** Wound healing assay in DU-145 and PC-3 after WFDC2 upregulation. The scale bar is 150 μm. **g** Immunoblot assay of EMT-related proteins in DU-145 and PC-3 after WFDC2 upregulation. **h** Immunofluorescence staining of WFDC2, p-EGFR, E-cadherin, and Vimentin in DU-145 and PC-3 after WFDC2 upregulation. **i** Diagram of mouse tail vein injection with PC-3-GFP vector or PC-3-GFP WFDC2 overexpressed. **j** Representative animal imaging and matching X-rays 6 weeks after tail vein injection of PC-3-GFP vector or PC-3-GFP WFDC2 overexpressed. *p < 0.05, **p < 0.01, ***p < 0.001; two-tailed Student’s *t*-test. The scale bars are 10 mm and 4 mm, respectively.

The bioinformatics analysis showed that WFDC2 is downregulated in metastatic PCa, and has a negative correlation with metastatic PCa and EMT (Fig. 2a, b). GSEA showed that WFDC2 was associated with the metastatic phenotype in various cancers (Supplementary Fig. 6a, b). To further demonstrate the relationship between WFDC2 and PCa metastasis, we performed metastasis-related experiments in vitro and in vivo. The transwell assay revealed that overexpression of WFDC2 significantly reduced the migration capacity of PC-3 and DU-145 cell lines (Fig. 2c, d). The wound healing assay indicated the same effect on inhibiting PCa metastasis (Fig. 2e, f). Consistently, overexpression of WFDC2 significantly downregulated the expression of N-Cad, Vimentin, and Snail, and upregulated the expression of E-Cad in PC-3 and DU-145 cell lines by immunoblot and immunofluorescence staining (Fig. 2g, h). PC-3 cells were injected into the tail vein of 8-week-old NOD/SCID mice (Fig. 2i). Compared with the vector group, the fluorescence intensity of PCa metastasis in the WFDC2 upregulated group was significantly decreased (Fig. 2j).

### WFDC2 inhibits EGFR activation in prostate cancer

**Fig. 3:**
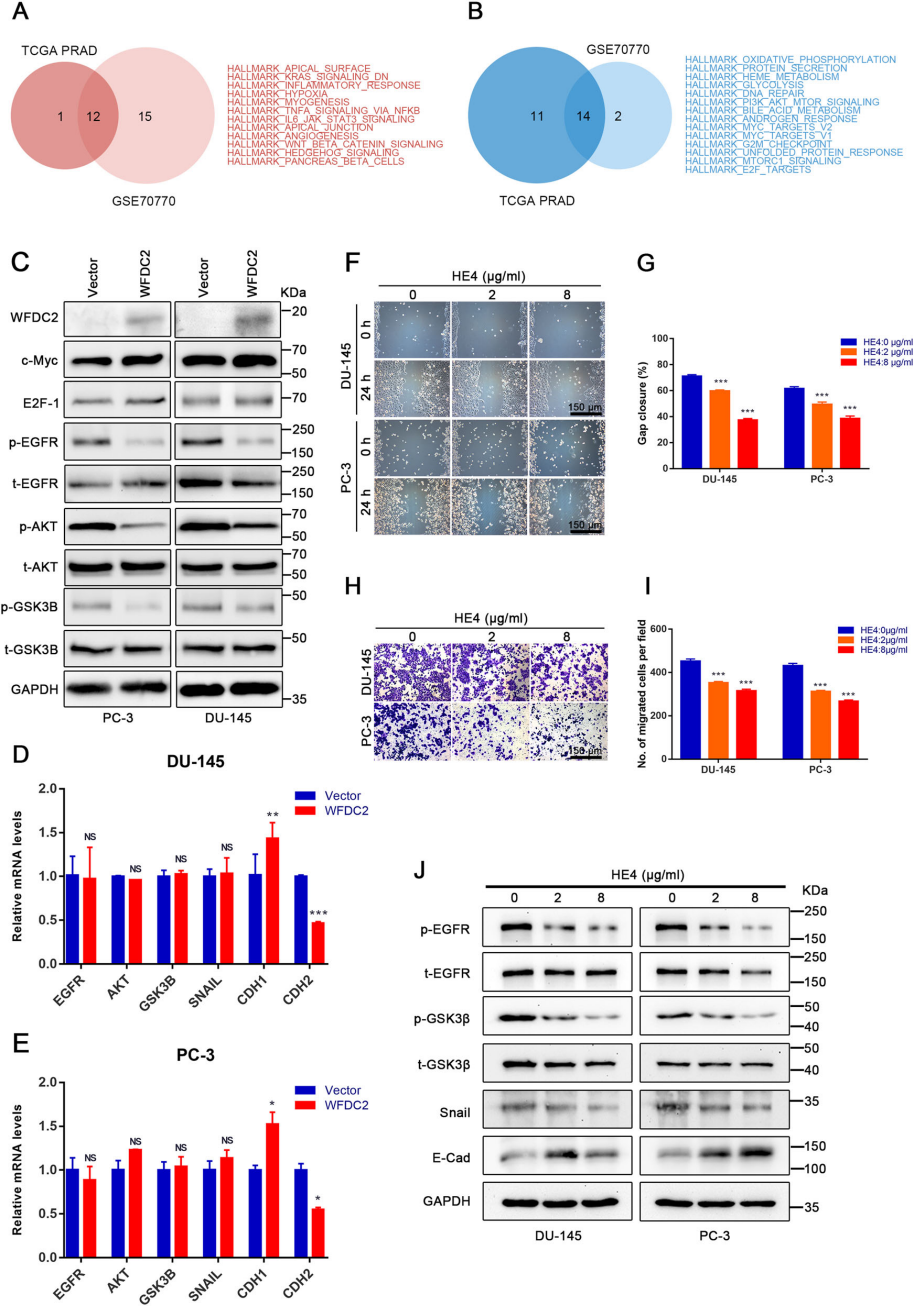
Overexpression of WFDC2 and addition of recombinant protein HE4 restrain the progression of EMT by inactivating the EGFR/AKT/GSK3B/Snail signaling pathway. **a**, **b** The 12 positively correlated pathways and 14 negatively correlated pathways after overlapping GSE70770 and TCGA. **c** Immunoblot assay of EGFR/AKT/GSK3B/Snail signaling protein in DU-145 and PC-3 after WFDC2 upregulation. **d**, **e** The relative mRNA expression of EGFR, AKT, GSK3β, Snail, CDH1, and CDH2 in DU-145 and PC-3 after WFDC2 upregulation. **f**, **g** Wound healing assay in DU-145 and PC-3 after adding recombinant protein HE4. The scale bar is 150 μm. **h**, **i** Transwell assay in DU-145 and PC-3 after adding recombinant protein HE4. The scale bar is 150 μm. **j** Immunoblot assay of p-EGFR, t-EGFR, p-GSK3β, t-GSK3β, Snail, and E-cadherin in DU-145 and PC-3 after adding recombinant protein HE4. *p < 0.05, **p < 0.01, ***p < 0.001; two-tailed Student’s *t*-test.

The GSVA of GSE70770 and TCGA show the WFDC2-related signaling pathway in Supplementary Fig. 7a, b. After overlapping GSE70770 and TCGA pathways, 14 negatively correlated pathways and 12 positively correlated pathways were listed in Fig. 3a, b. We detected some proteins in these pathways after upregulating WFDC2. The expression of c-Myc and E2F-1 showed no significant change. Interestingly, we found that the phosphorylation of EGFR, AKT, and GSK3B were downregulated in PC-3 and DU-145 cell lines (Fig. 3c). The downregulation of p-EGFR was also proven by immunofluorescence staining (Fig. 2h). After overexpression of WFDC2, the mRNA levels of EGFR, AKT, GSK3B, and Snail were not significantly changed in PC-3 and DU-145 cell lines (Fig. 3d, e). Notably, the mRNA level of CDH1 was obviously upregulated, and CDH2 was upregulated after WFDC2 upregulated in PC-3 and DU-145 cell lines (Fig. 3d, e).

To determine whether WFDC2 affects PCa metastasis intracellularly or extracellularly, the recombinant protein HE4 was added to the cell culture medium of PC-3 and DU-145. Transwell and wound healing assays indicated that 2 μg/ml and 8 μg/ml of HE4 significantly inhibited PCa metastasis compared with the control group (Fig. 3f–i). In addition, 2 μg/ml and 8 μg/ml of HE4 downregulated the protein level of p-EGFR, p-GSK3B, and Snail and upregulated the protein level of E-cad by immunoblot assay (Fig. 3j).

### WFDC2 binds to the EGFR extracellular domain

**Fig. 4:**
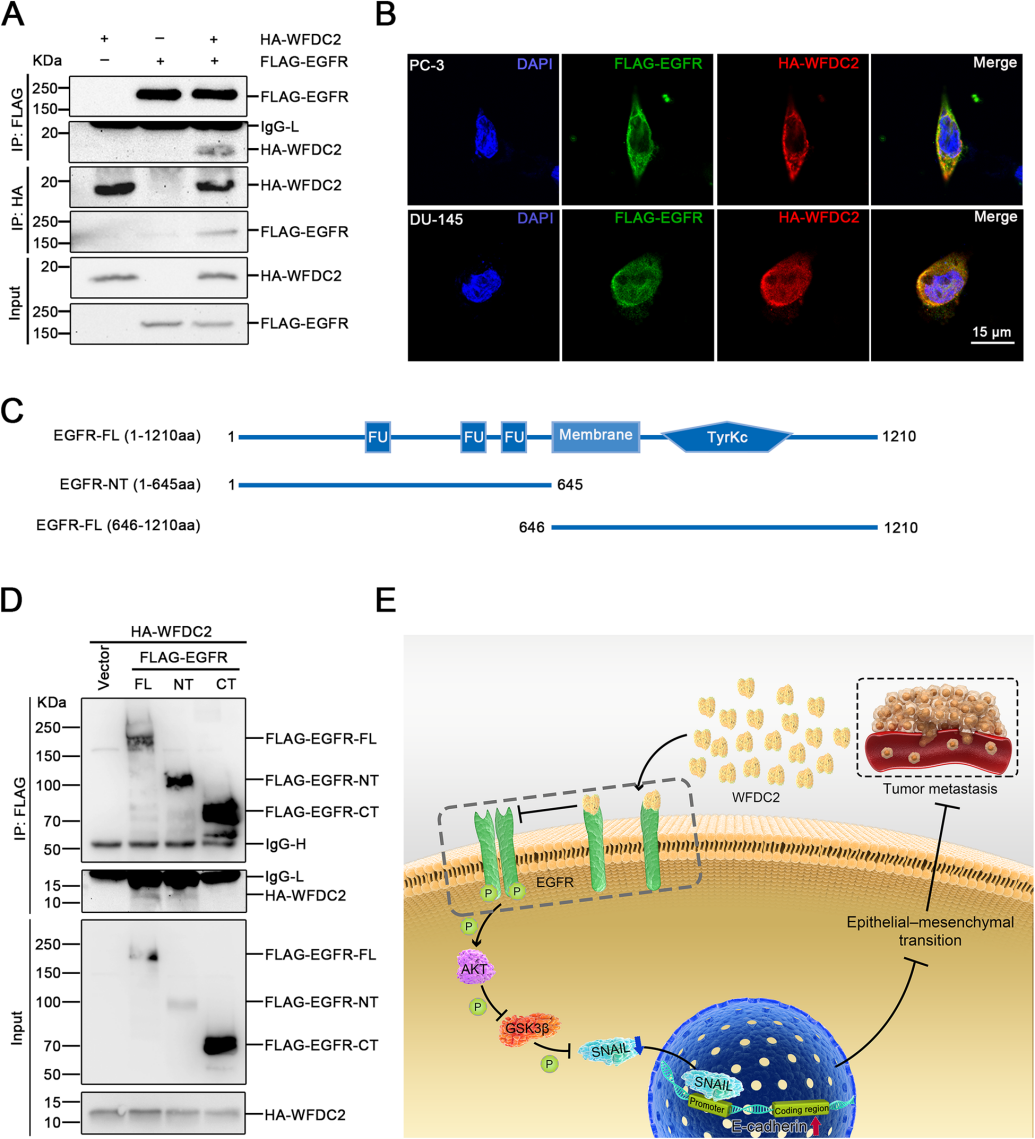
WFDC2 binds to the EGFR extracellular domain. **a** Co-IP assay of WFDC2 and EGFR in 293-T cell line. **b** Co-localization assay of WFDC2 and EGFR in DU-145 and PC-3 cell lines. The scale bar is 15 μm. **c** Diagram of EGFR-FL, EGFR-NT, and EGFR-CT. **d** Co-IP assay between WFDC2 and EGFR-FL, EGFR-NT, and EGFR-CT in 293-T cell line. **e** Graphic model of WFDC2 suppressed prostate cancer metastasis by inactivating EGFR signaling.

To explain the mechanism of these protein changes, we performed co-IP analysis of WFDC2 and EGFR. Encouragingly, the results revealed that WFDC2 interacted with EGFR in 293 T cell line (Fig. 4a). We also performed immunofluorescence staining in PC-3 and DU-145 cell lines. Likewise, the results proved that WFDC2 colocalized with EGFR in the plasma membrane (Fig. 4b). To further validate the specific interaction domain between WFDC2 and EGFR in prostate cancer, we divided EGFR into the extracellular domain EGFR-NT and the intracellular domain EGFR-NT (Fig. 4c). The results showed that WFDC2 interacted with the extracellular domain of EGFR (Fig. 4d). Taken together, our results suggest that the secreted protein WFDC2 binds to the extracellular domain of EGFR to inhibit the activation of EGFR and then inhibits PCa metastasis (Fig. 4e)

### Recombinant protein HE4 suppresses EMT in prostate cancer by inactivating EGFR signaling

**Fig. 5:**
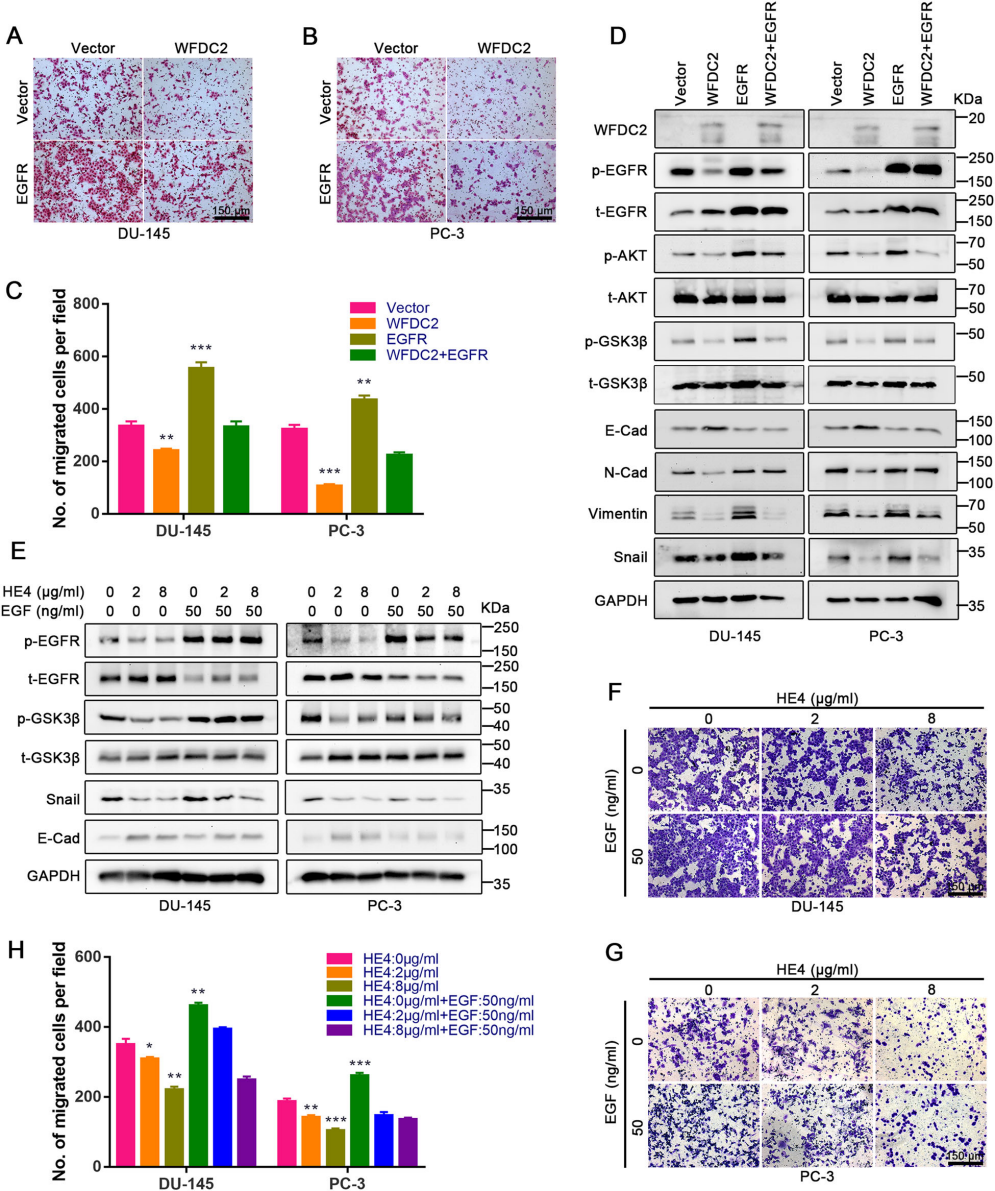
EGFR and EGF can rescue the metastatic ability repressed by WFDC2. **a**–**c** Transwell assay in DU-145 and PC-3 after overexpressing EGFR and WFDC2. The scale bar is 150 μm. **d** Immunoblot assay of EGFR-related and EMT-related proteins in DU-145 and PC-3 after overexpressing EGFR and WFDC2. **e** Immunoblot assay of EGFR-related and EMT-related proteins in DU-145 and PC-3 after adding EGF and HE4. **f**–**h** Transwell assay in DU-145 and PC-3 after adding EGF and HE4. *p < 0.05, **p < 0.01, ***p < 0.001; two-tailed Student’s *t*-test. The scale bar is 150 μm.

After demonstrating that WFDC2 inhibits the metastatic ability of prostate cancer by binding to the EGFR extracellular domain, we overexpressed WFDC2 and EGFR in PC-3 and DU-145 simultaneously. Transwell assay showed that EGFR can significantly rescue prostate cancer metastasis inhibited by WFDC2 (Fig. 5a–c). Furthermore, the expression of EGFR downstream proteins and EMT-related proteins was also restored by EGFR overexpression (Fig. 5d). EGF, a widely verified ligand of EGFR, activates EGFR phosphorylation by binding to the EGFR extracellular domain. Considering that HE4 and EGF bind to the same domain of EGFR, we added the recombinant proteins HE4 and EGF to PC-3 and DU-145 simultaneously. Notably, the results showed that EGF can significantly rescue HE4 mediated the prostate cancer metastasis suppression, which is consistent with the effect of EGFR overexpressed (Fig. 5e–h). Furthermore, the relative mRNA expression of WFDC2, EGFR, AKT, Snail, CDH1, and CDH2 in DU-145 and PC-3 after WFDC2 upregulation was shown in Supplementary Fig. 8a–d. To further verify these results, the recombinant protein HE4 and EGFR knockdown were used in PC-3 and DU-145. Transwell assay showed the metastasis ability inhibited by recombinant protein HE4 was significantly reduced after EGFR knockdown (Supplementary Fig. 8e, f). Immunoblot assay also revealed that the protein level of E-cad, N-cad, Vimentin, and Snail had a slight but not significant change between the HE4: 0 μg/ml group and the HE4: 8 μg/ml group after EGFR knockdown (Supplementary Fig. 8g). These results showed that recombinant protein HE4 suppresses EMT in prostate cancer by inactivating EGFR signaling.

### WFDC2 is an independent and favorable prognostic factor of human prostate cancer

**Fig. 6:**
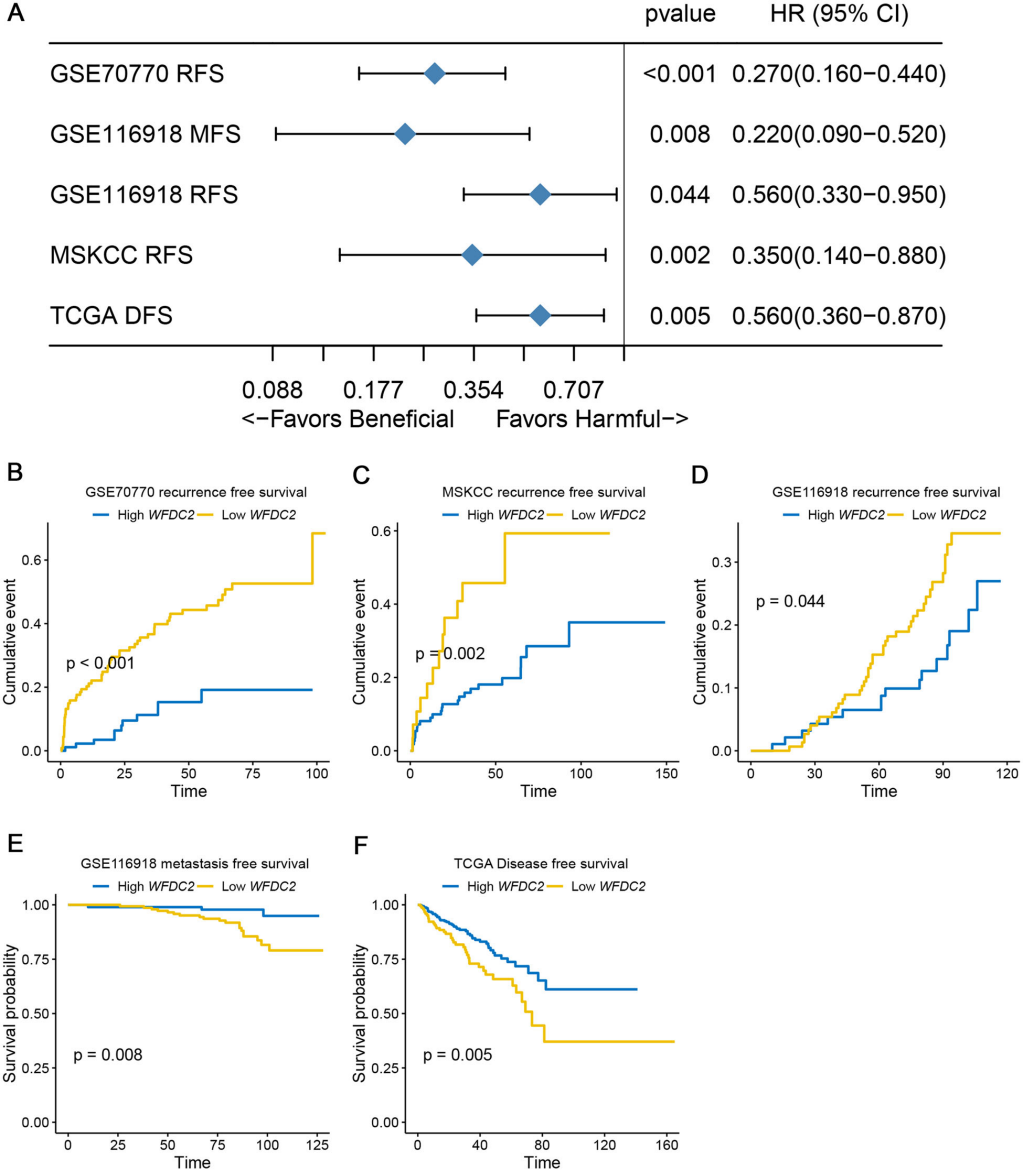
Prognostic value validation via different datasets. **a** Forest plot summary of the log-rank analysis of WFDC2 in different datasets. The blue diamond squares on the transverse lines represent the HR, and the black transverse lines represent the 95% CI. The *p* value and 95% CI for each dataset are displayed in detail. **b** Recurrence-free survival (RFS) of WFDC2 in GSE70770. **c** RFS of WFDC2 in MSKCC cohort. **d** RFS of WFDC2 in GSE116918. **e** Metastasis-free survival of WFDC2 in GSE116918. **f** Disease-free survival of WFDC2 in TCGA-PRAD.

Here, the optimal cutoff point was selected for the survival analysis in each dataset. According to the optimal cutoff, patients were stratified into low- and high-expression groups. The forest plots showed that higher expression of WFDC2 was significantly associated with better prognosis in RFS, MFS, and DFS (Fig. 6a). The KM curves presented RFS of GSE70770 (*p* value < 0.001, Fig. 6b), RFS of MSKCC (*p* value = 0.002, Fig. 6c), RFS of GSE116918 (*p* value = 0.044, Fig. 6d), MFS of GSE116918 (*p* value = 0.008, Fig. 6e), and DFS of TCGA-PRAD (*p* value = 0.005, Fig. 6f). These results indicated WFDC2 might serve as a favorable prognostic biomarker in prostate cancer patients.

## Discussion

As the tumor with the highest incidence in men, most men will develop prostate cancer when live long enough^22^. Androgen-dependent prostate cancer (ADPC) is sensitive to ADT. Unfortunately, most ADPC eventually progresses to castration-resistant prostate cancer (CRPC)^23^. The prognosis of CRPC is very poor and treating CRPC is very difficult. In addition, the metastatic ability of CRPC is worse than that of ADPC. Once CRPC develops a distant metastasis, the current treatment is not very effective. Therefore, a large number of studies have attempted to find a mechanism related to CRPC metastasis to find a more effective treatment for CRPC^24,25^.

EGFR is a member of the ERBB family of transmembrane receptor tyrosine kinases^26^. The ligands (EGF, ANG, EREG, EPGN, and TGFA) can bind to the extracellular domain of EGFR and trigger homo- and/or heterodimerization, and autophosphorylation of EGFR^27–31^. EGFR has been used as an effective therapeutic target for tyrosine kinase inhibitors in lung and pancreatic cancers^32,33^. Furthermore, the activation of EGFR to promote tumor metastasis has been widely proven in various tumors^34–36^.

Our study first screened the diagnostic marker WFDC2 by WGCNA. After validation by database and tissue microarray, we found that WFDC2 was dramatically downregulated in human prostate cancer and negatively correlated with Gleason score. We also uncovered that WFDC2 is an independent and favorable prognostic factor of human prostate cancer. Interestingly, WFDC2 has been extensively studied in ovarian cancer and widely used as a clinical diagnostic marker for ovarian cancer. The huge difference in WFDC2 between prostate cancer and ovarian cancer is of great interest to us.

In our study, we found that WFDC2 was significantly overexpressed in nonmetastatic PCa compared with metastatic PCa. Therefore, we speculated that WFDC2 may be negatively correlated with prostate cancer metastasis. In the PCa cell lines PC-3 and DU-145, we demonstrated that WFDC2 can significantly inhibit PCa metastasis through transwell and wound healing assays in vitro and a metastatic model in vivo. Furthermore, we have revealed that WFDC2 can significantly inhibit the metastasis of PCa by suppressing the activation of EGFR/AKT/GSK3B pathways.

Considering that WFDC2 is a small secreted protein, we speculated that WFDC2 may interact with the extracellular domain of cell membrane receptors to affect PCa metastasis. To further validate this hypothesis, we added the recombinant protein HE4 to the culture media of PC-3 and DU-145, and the effect of HE4 on PCa metastasis was consistent with that of WFDC2 upregulation. In addition, co-IP and co-localization experiments were performed, and we found that WFDC2 can bind to the extracellular domain of EGFR to influence the activation of EGFR. Considering that HE4 and EGF bind to the same domain of EGF, we speculated that HE4 and EGF may competitively bind the extracellular domain of EGFR, and subsequently suppress EGFR activation and PCa metastasis.

In conclusion, our study validated a novel metastasis-related gene in prostate cancer. WFDC2, which may serve as a potential clinical treatment target of PCa, suppressed prostate cancer metastasis by inactivating EGFR signaling.

## Supplementary information

Supplementary information

Supplementary information2

Supplementary information3

Supplementary information4

Supplementary information5

Supplementary information6

Supplementary information7

Supplementary information8

Supplementary information9

Supplementary information10
