# Supplementary material for: WFDC2 suppresses prostate cancer metastasis by modulating EGFR signaling inactivation
Source: Cell Death Dis. 2020 Jul 16;11(7):537. doi: 10.1038/s41419-020-02752-y (PMC7366654; doi:10.1038/s41419-020-02752-y)
Supplement: Supplementary file 1 — Supplementary information [file 41419_2020_2752_MOESM1_ESM.docx]

**Supplementary Figures legends**

**Supplementary Figure S1. Outlier detection for GSE70770.** Sample clustering via average clustering method to remove outlier samples in GSE70770.

**Supplementary Figure S2. Identification of WFDC2 as a critical tumor suppressor of human prostate cancer via WGCNA analysis.** **(A)** Sample cluster of GSE70770. **(B)** Assessment of soft-thresholding power in the WGCNA analysis. Analysis of the scale-free fit index and the mean connectivity for various soft-thresholding powers (upper). Histogram of connectivity distribution and the scale-free topology (down). **(C)** Dynamic tree cut. Dendrogram of all selected genes clustered by a dissimilarity measure (1-TOM). **(D)** Heatmap showing the relationship between tumor percentage (%) and module eigengenes. **(E)** Distribution of average gene signiﬁcance and errors in the modules associated with tumor percentage (%). **(F)** WFDC2 expression level in different cancers based on Oncomine database. **(G)** Expression of WFDC2 in prostate cancer and non-tumor.

**Supplementary Figure S3. Diagnostic value validation via Human protein atlas database.** **(A)** Dotplot of the protein expression level of WFDC2 in various human normal tissues. **(B)** Dotplot of the protein expression level of WFDC2 in various human tumor tissues. **(C)** IHC staining of WFDC2 in prostate cancer tissues and normal prostate tissues. The scale bar is 400 μm.

**Supplementary Figure S4. The clinical information of tissue microarray.** **(A)** Overview of tissues microarray. The scale bar is 800 μm. **(B)** Gleason score, tissue type and staining intensity of tissue microarray.

**Supplementary Figure S5. WFDC2 has no effect on proliferation and apoptosis of prostate cancer. (A)** MTT assay in DU-145 and PC-3 after WFDC2 upregulation. **(B)** Flow cytometry analysis of cell cycle in DU-145 and PC-3 after WFDC2 upregulation. **(C)** Flow cytometry analysis of apoptosis in DU-145 and PC-3 after WFDC2 upregulation.

**Supplementary Figure S6. WFDC2 is highly negatively correlated with metastasis phenotype in various cancers. (A)** Barplot to show GSEA results associated with metastasis phenotype in various cancers. Color scale bar represented the p value and color bars represented different types of cancer. **(B)** GSEA plot related to prostate cancer metastasis.

**Supplementary Figure S7.** **The GSVA of WFDC2 in GSE70770 and TCGA show the WFDC2 related signaling pathway in.** **(A)** The GSVA of WFDC2 in GSE70770. **(B)** The GSVA of WFDC2 in TCGA.

**Supplementary Figure S8.** **The relative mRNA expression after overexpressing WFDC2 and EGFR.** **(A, B)** The relative mRNA expression of WFDC2 and EGFR after overexpressing WFDC2 and EGFR. **(C, D)** The relative mRNA expression after overexpressing WFDC2 and EGFR. **(E, F)** Transwell assay in DU-145 and PC-3 after adding the recombinant protein HE4 and siEGFR. The scale bar is 150 μm. **(G)** Immunoblot assay of EMT-related proteins in DU-145 and PC-3 after adding the recombinant protein HE4 and siEGFR. *p < 0.05, **p < 0.01, ***p < 0.001; two-tailed Student’s t-test.
