# Supplementary material for: WFDC2 suppresses prostate cancer metastasis by modulating EGFR signaling inactivation
Source: Cell Death Dis. 2020 Jul 16;11(7):537. doi: 10.1038/s41419-020-02752-y (PMC7366654; doi:10.1038/s41419-020-02752-y)
Supplement: Supplementary file 2 — Supplementary information2 [file 41419_2020_2752_MOESM2_ESM.docx]

**Supplementary Tables**

**Supplementary Table 1. List of primers for plasmid construction.**

| Gene | Forward primer | Reverse prime |
| --- | --- | --- |
| WFDC2 | AATGGATCCATGCCTGCTTGTCGC | GGCCTCGAGTCAGAAATTGGGAGT |
| EGFR-FL | AAAAAGCTTATGCGACCCTCCGGG | AAAACCGGTTCATGCTCCAATAAA |
| EGFR-NT | AAAAAGCTTATGCGACCCTCCGGG | AAAACCGGTGGACGGGATCTTAGG |
| EGFR-CT | AAAAAGCTTATGATCGCCACTGGG | AAAACCGGTTGCTCCAATAAATTC |

**Supplementary Table 2. List of primers for qRT-PCR.**

| Gene | Forward primer | Reverse prime |
| --- | --- | --- |
| WFDC2 | AGAACTGCACGCAAGAGTG | TTGAGGTTGTCGGCGCATT |
| EGFR | AGGCACGAGTAACAAGCTCAC | ATGAGGACATAACCAGCCACC |
| AKT | AGCGACGTGGCTATTGTGAAG | GCCATCATTCTTGAGGAGGAAGT |
| GSK3B | GGCAGCATGAAAGTTAGCAGA | GGCGACCAGTTCTCCTGAATC |
| Snail | TCGGAAGCCTAACTACAGCGA | AGATGAGCATTGGCAGCGAG |
| CDH1 | CGAGAGCTACACGTTCACGG | GGGTGTCGAGGGAAAAATAGG |
| CDH2 | TCAGGCGTCTGTAGAGGCTT | ATGCACATCCTTCGATAAGACTG |
| GAPDH | GGAGCGAGATCCCTCCAAAAT | GGCTGTTGTCATACTTCTCATGG |
