# Supplementary figures and images for: WFDC2 suppresses prostate cancer metastasis by modulating EGFR signaling inactivation
Source: Cell Death Dis. 2020 Jul 16;11(7):537. doi: 10.1038/s41419-020-02752-y (PMC7366654; doi:10.1038/s41419-020-02752-y)

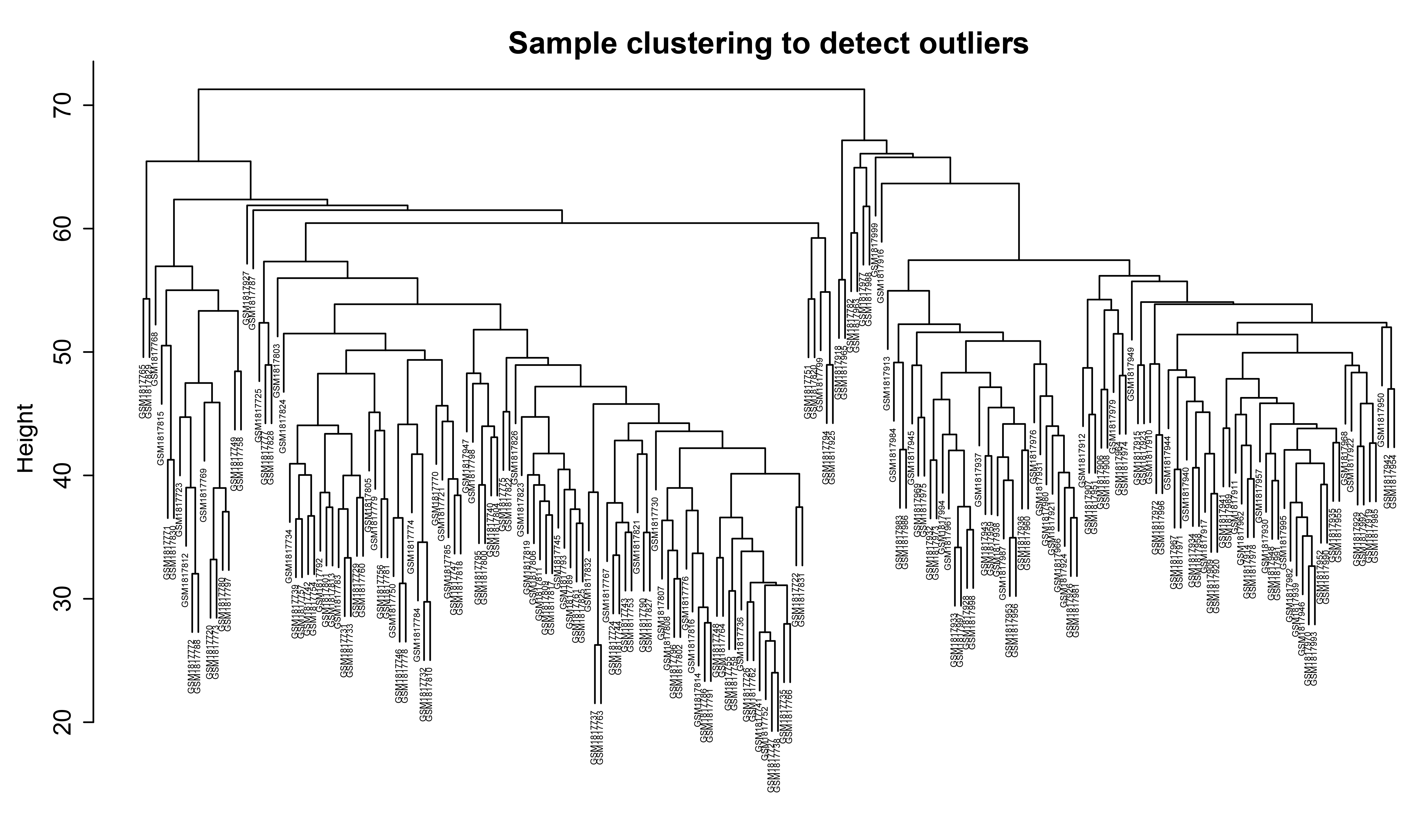

Supplement: Supplementary file 3 — Supplementary information3 [file 41419_2020_2752_MOESM3_ESM.tif]

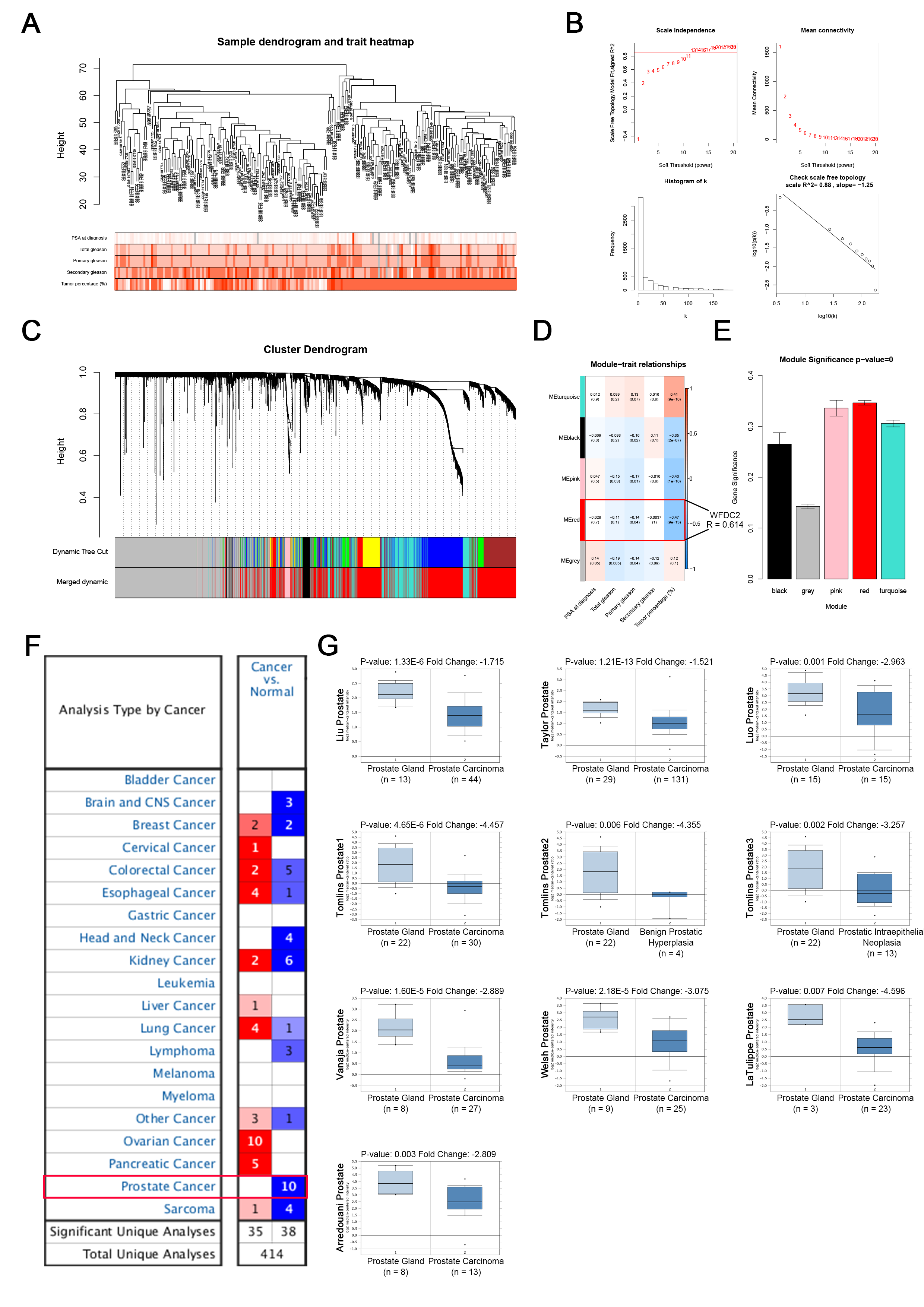

Supplement: Supplementary file 4 — Supplementary information4 [file 41419_2020_2752_MOESM4_ESM.tif]

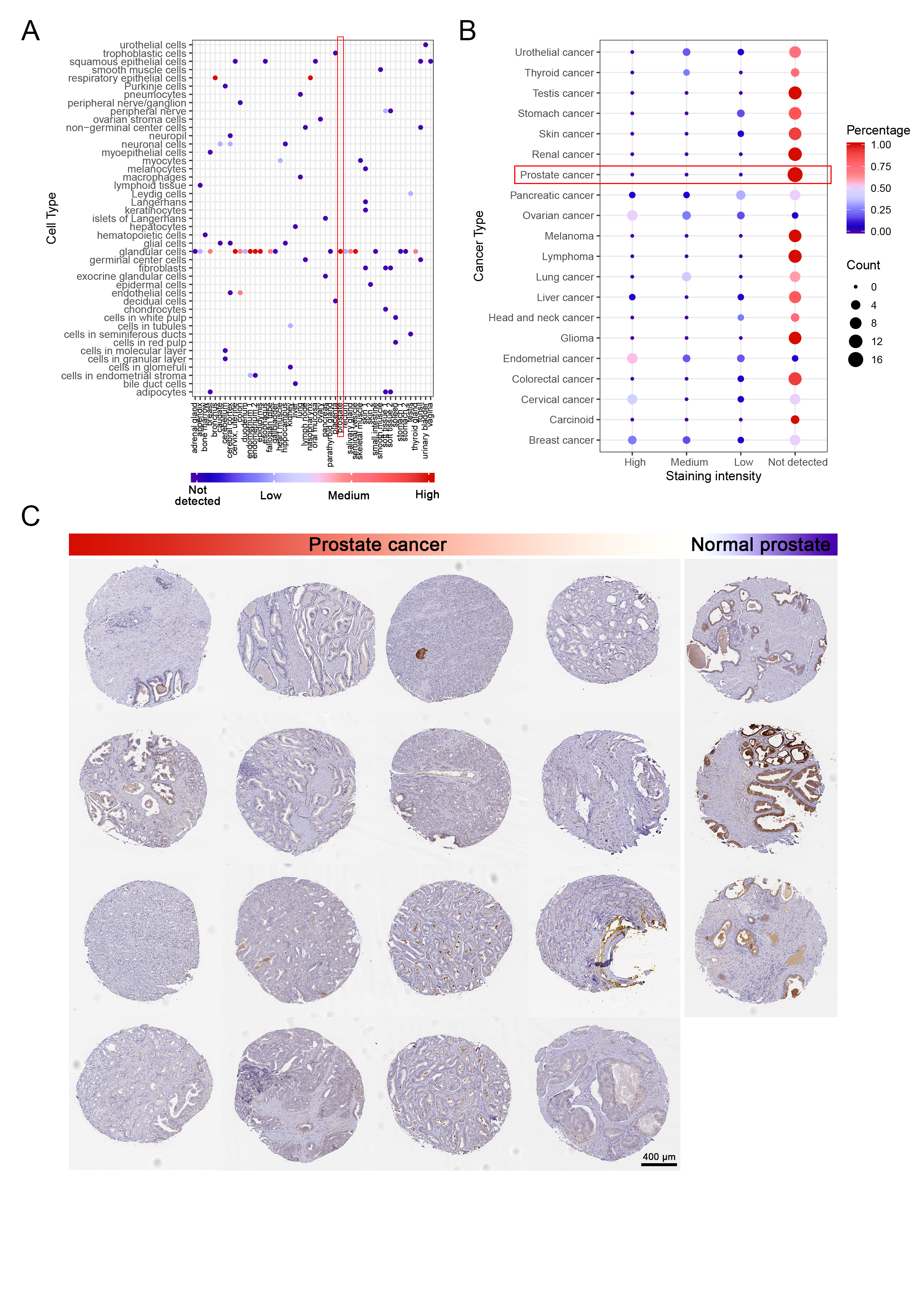

Supplement: Supplementary file 5 — Supplementary information5 [file 41419_2020_2752_MOESM5_ESM.tif]

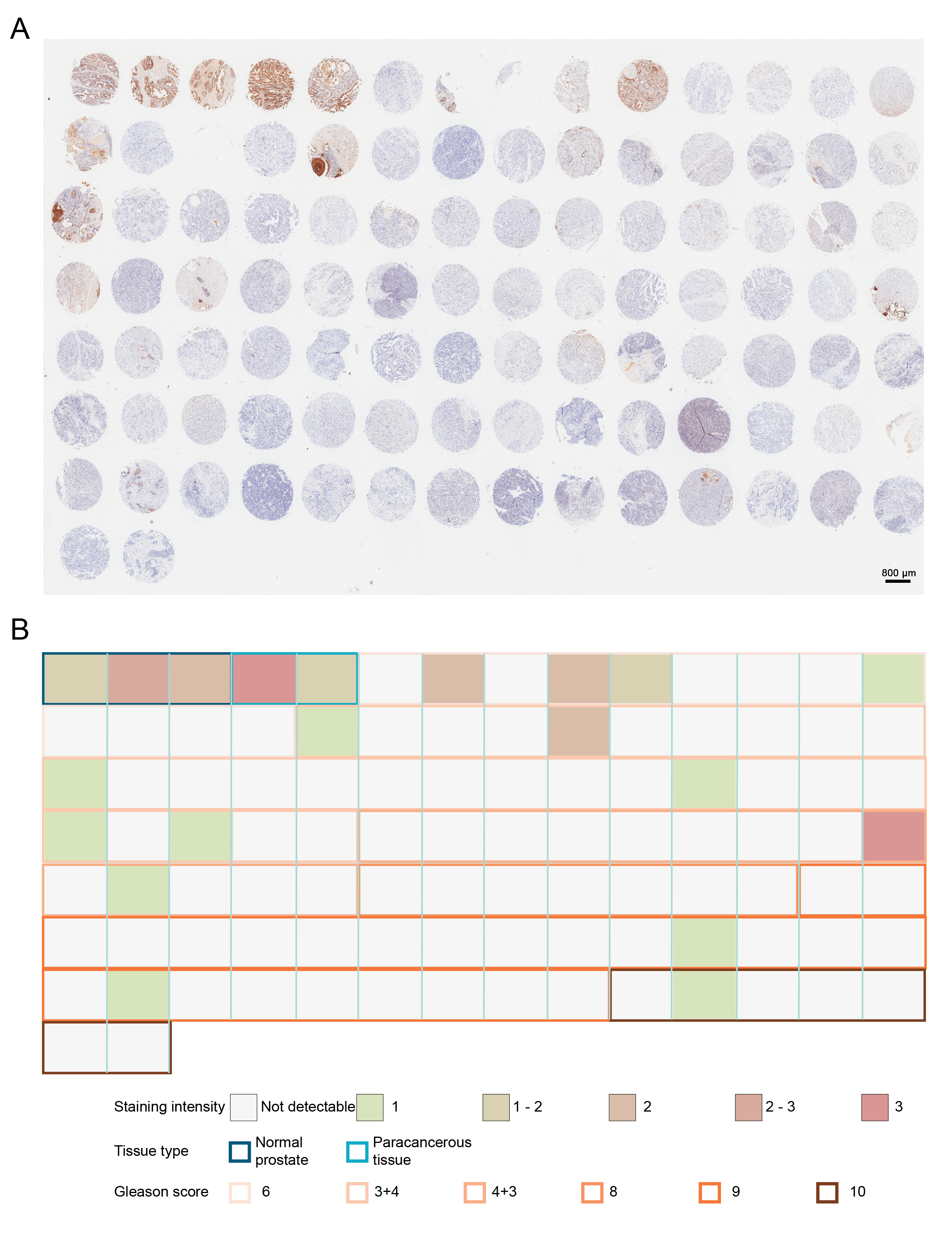

Supplement: Supplementary file 6 — Supplementary information6 [file 41419_2020_2752_MOESM6_ESM.tif]

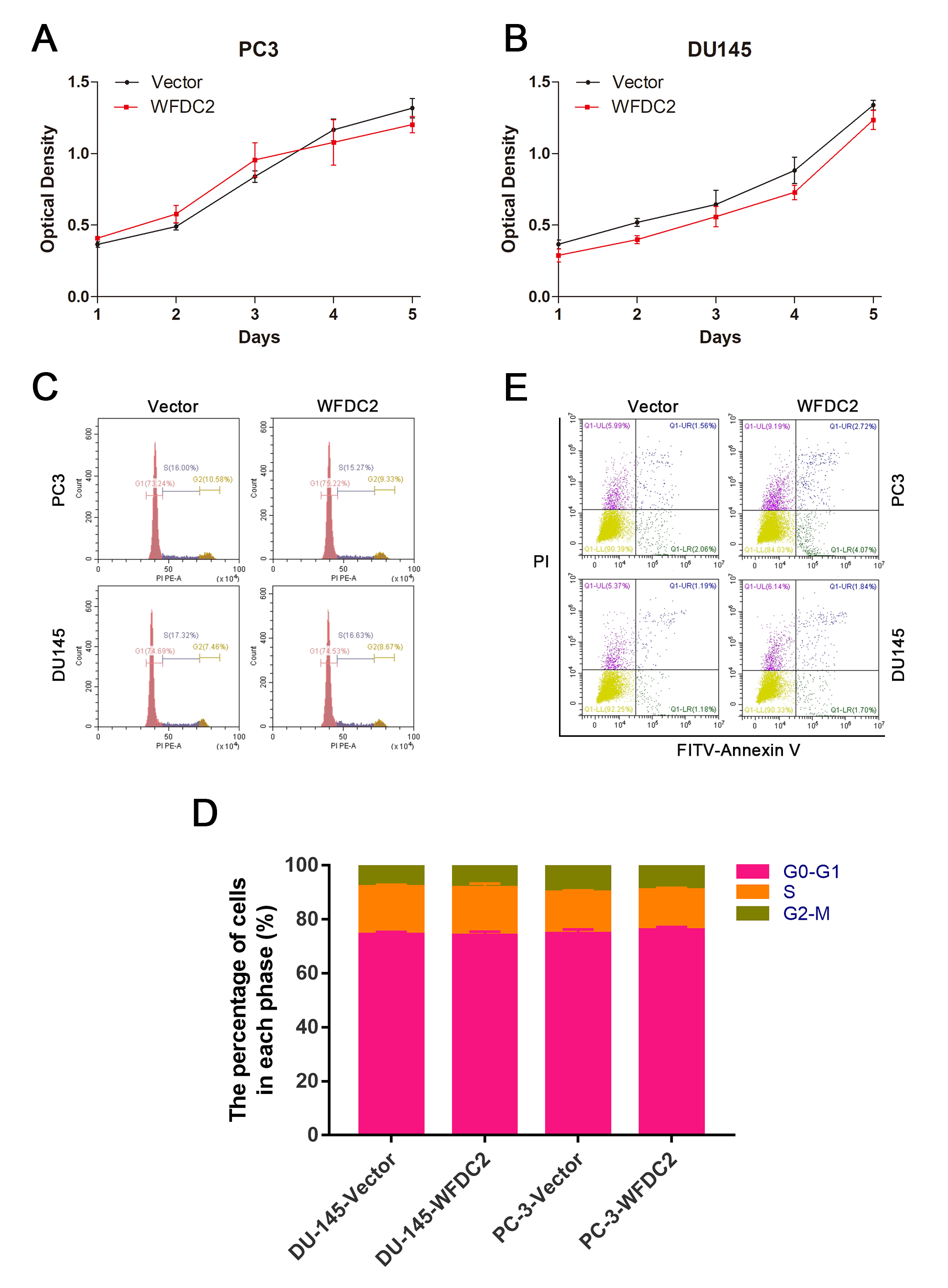

Supplement: Supplementary file 7 — Supplementary information7 [file 41419_2020_2752_MOESM7_ESM.tif]

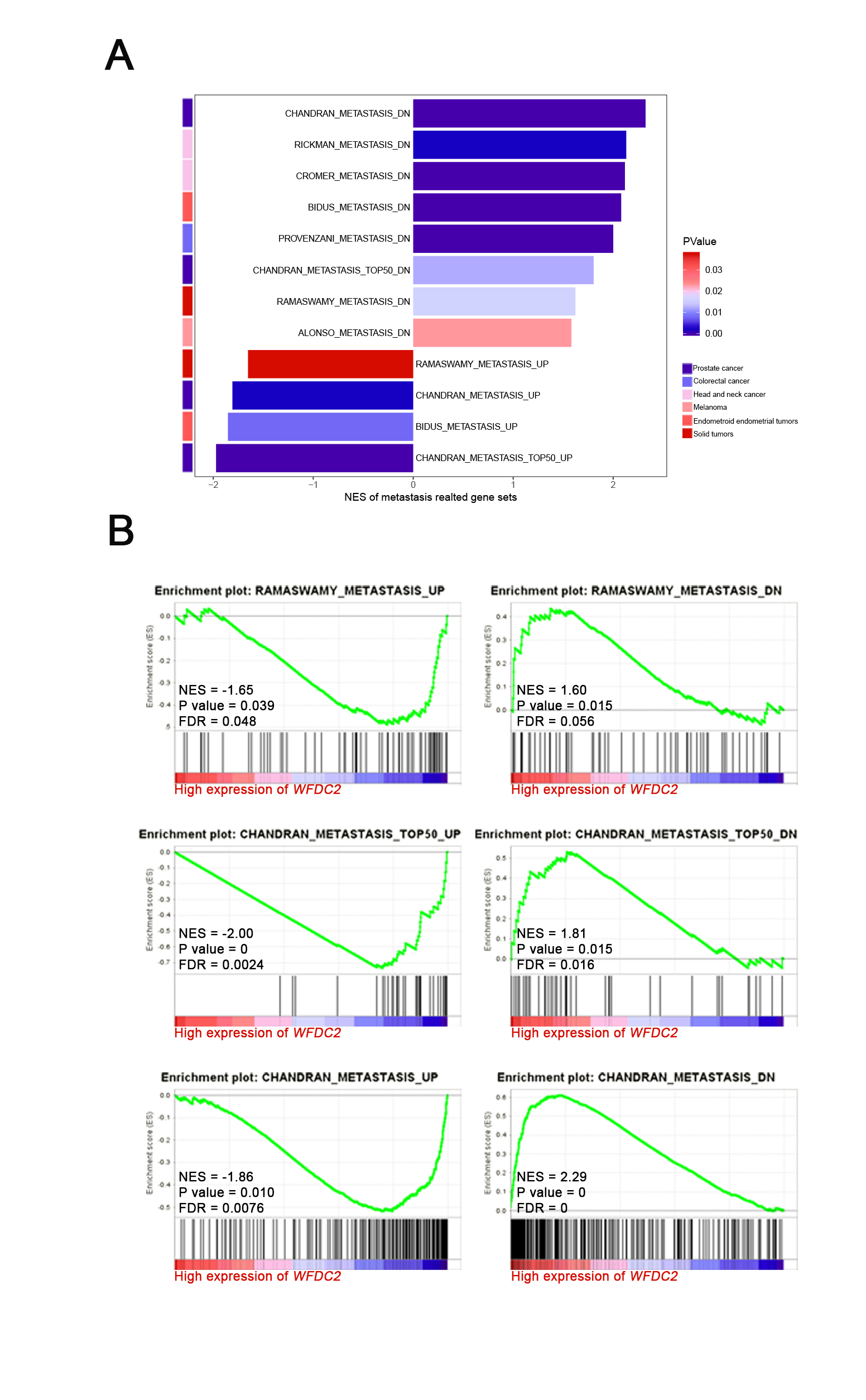

Supplement: Supplementary file 8 — Supplementary information8 [file 41419_2020_2752_MOESM8_ESM.tif]

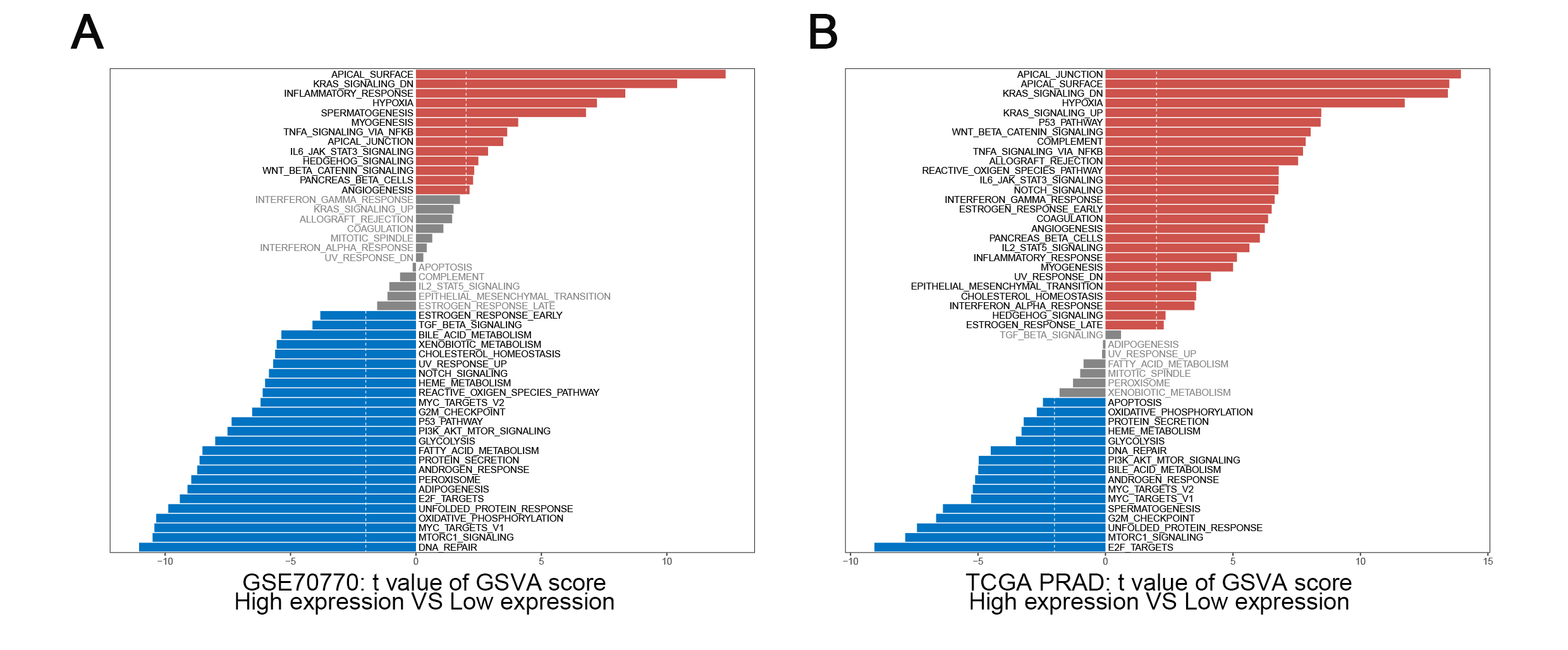

Supplement: Supplementary file 9 — Supplementary information9 [file 41419_2020_2752_MOESM9_ESM.tif]

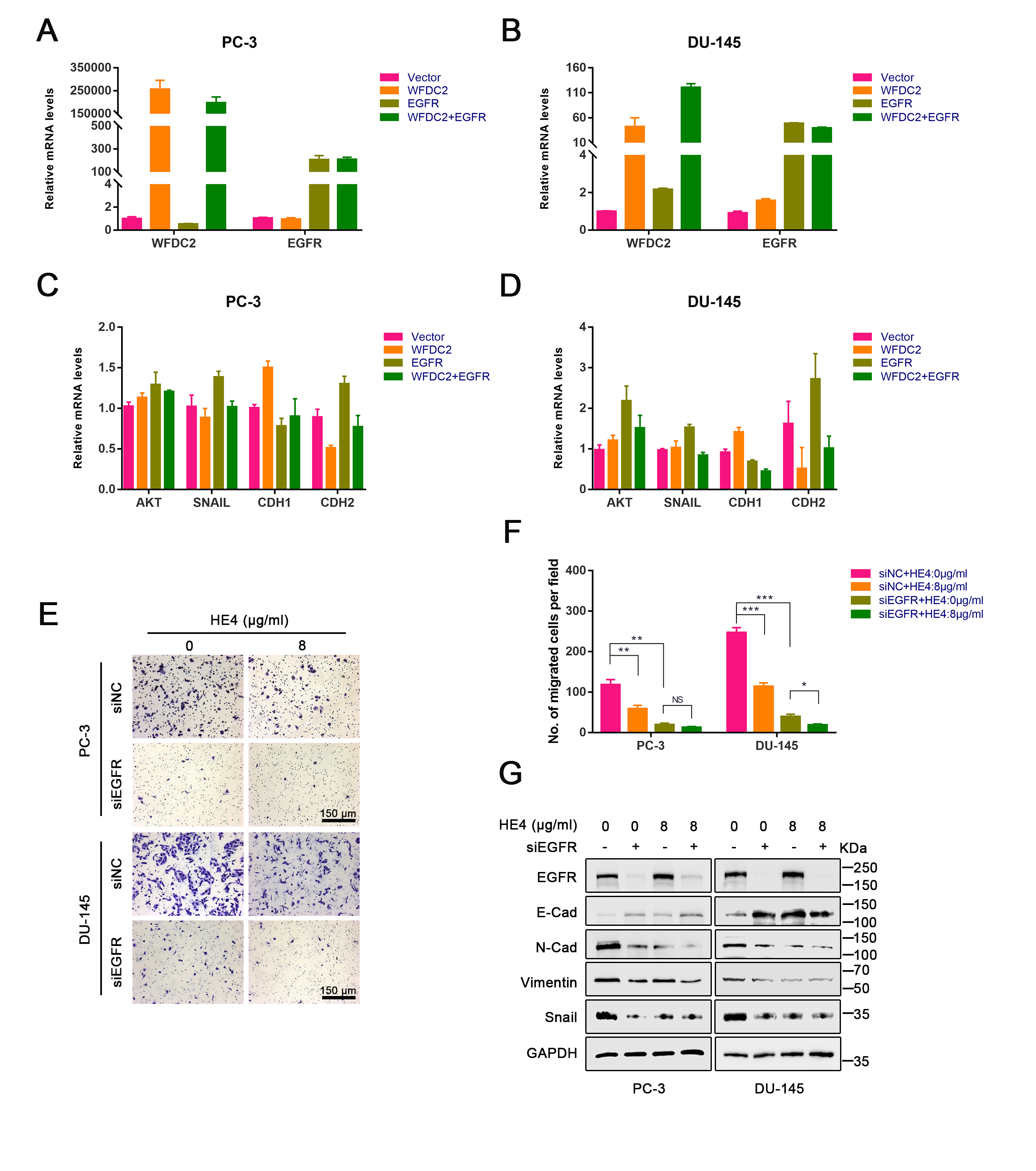

Supplement: Supplementary file 10 — Supplementary information10 [file 41419_2020_2752_MOESM10_ESM.tif]
